# Cortisol Promotes Surface Translocation of *Porphyromonas gingivalis*

**DOI:** 10.3390/pathogens11090982

**Published:** 2022-08-27

**Authors:** Hey-Min Kim, Christina Magda Rothenberger, Mary Ellen Davey

**Affiliations:** Department of Oral Biology, College of Dentistry, University of Florida, Gainesville, FL 32610, USA

**Keywords:** cortisol, surface translocation, *Porphyromonas gingivalis*, T9SS

## Abstract

Studies are showing that the stress hormone cortisol can reach high levels in the gingival sulcus and induce shifts in the metatranscriptome of the oral microbiome. Interestingly, it has also been shown that cortisol can influence expression levels of Type IX Secretion System (T9SS) genes involved in gliding motility in bacteria belonging to the phylum Bacteroidota. The objective of this study was to determine if cortisol impacts gene expression and surface translocation of *Porphyromonas gingivalis* strain W50. To conduct these experiments, *P. gingivalis* was stabbed to the bottom of soft agar plates containing varying cortisol concentrations (0 μM, 0.13 μM, 1.3 μM, and 13 μM), and surface translocation on the subsurface was observed after 48 h of incubation. The results show that when grown with certain nutrients, i.e., in rich medium with the addition of sheep blood, lactate, or pyruvate, cortisol promotes migration of *P. gingivalis* in a concentration-dependent manner. To begin to examine the underlying mechanisms, quantitative PCR was used to evaluate differential expression of genes when *P. gingivalis* was exposed to cortisol. In particular, we focused on differential expression of T9SS-associated genes, including *mfa5,* since it was previously shown that Mfa5 is required for cell movement and cell-to-cell interactions. The data show that *mfa5* is significantly up-regulated in the presence of cortisol. Moreover, an *mfa5* deletion mutant showed less surface translocation compared to the wild-type *P. gingivalis* in the presence of cortisol, and the defects of the *mfa5* deletion mutant were restored by complementation. Overall, cortisol can stimulate *P. gingivalis* surface translocation and this coincides with higher expression levels of T9SS-associated genes, which are known to be essential to gliding motility. Our findings support a high possibility that the stress hormone cortisol from the host can promote surface translocation and potentially virulence of *P. gingivalis*.

## 1. Introduction

The collective pathogenic potential of an oral microbial community depends upon the susceptibility of the host, the composition of the community, and the outcome of interbacterial interactions [1]. Host susceptibility to periodontitis is the transition from host–microbe symbiosis to dysbiosis and disease, and is determined by a variety of factors such as genetics, diet, stress, or behaviors such as smoking. Among the host factors, stress has been known as a significant risk factor for a variety of diseases including periodontal disease since it can impact the composition of the commensal microbiota in the human microbiome [2]. Recent studies have shown that stress hormones, including cortisol, can induce shifts in the gene expression profile of the oral microbiome [3,4,5].

Cortisol is the most abundant glucocorticoid in humans, and its levels in saliva and serum have been shown to increase with the severity of periodontal disease [6,7]. In the oral cavity, glucocorticoids including cortisol depress immunity by inhibiting the production of secretory immunoglobulins, and neutrophil functions, all of which may disrupt homeostasis [8].

*Porphyromonas gingivalis* is a Gram-negative anaerobic bacterium that is strongly associated with severe periodontitis [9,10,11]. Recent reports indicate that *P. gingivalis* infection is also linked to systemic inflammatory disorders such as diabetes, Alzheimer’s disease, cardiovascular disease, and rheumatoid arthritis [12,13,14,15,16,17,18]. Like other members of the phylum Bacteroidota, *P. gingivalis* has a type IX secretion system (T9SS) that is responsible for the secretion of over thirty *P. gingivalis* proteins bearing a specific C-terminal domain, including the trypsin-like gingipains, which are key virulence determinants [19]. Recently, it has been proposed that cortisol can regulate expression levels of T9SS-associated genes and biofilm formation in *Flavobacterium columnare*, an aquatic bacterium of the phylum Bacteroidota [20]. While other *Bacteroides* species display a T9SS-mediated gliding motility that is readily apparent on the surface of an agar plate [19,21], *P. gingivalis* does not display this type of motility. However, surface translocation at the interface of soft agar and a glass or plastic surface has been demonstrated and it was determined that the T9SS and fimbrial proteins are central to this process [22].

Importantly, the metabolism of *P. gingivalis* is atypical. This bacterium is highly proteolytic and asaccharolytic, utilizing protein substrates as the main source for energy production and proliferation [23,24,25,26]. In addition, metabolic coupling has been discovered that allows *P. gingivalis* to combine amino acid fermentation with the consumption of pyruvate and lactate to generate energy. This affects not only central carbon metabolism, but also the expression of fimbrial adhesins, a requirement for surface colonization and migration [27]. *P. gingivalis* produces two distinct fimbriae, major fimbriae and minor fimbriae, on its cell surface [28]. The major fimbriae are encoded by the *fim* gene cluster with *fimA* as the main polymerizing subunit, *fimB* as the anchor, and *fimC/D/E* as the tip proteins. Similarly, the minor fimbriae are encoded the *mfa* gene cluster with *mfa1* as the main polymerizing subunit, *mfa2* as the anchor, and *mfa3/4/5* as the tip proteins. Among the Mfa subunits, Mfa5 is unique in that it contains a C-terminal domain (CTD) that directs its secretion through the T9SS, and it also contains a von Willebrand factor type A (vWF) domain, which is widely distributed among archaea, bacteria, and eukaryotes [29,30]. vWF domains have been studied in integrins, extracellular matrix proteins, and magnesium chelatases and are known to perform diverse functions, yet vWF domains are primarily involved in protein–protein interactions or adhesion [31]. In bacteria, vWF-containing proteins at the tips of fimbrial fibers in a few Gram-positive pathogenic bacteria have been reported to play important roles in attachment to host cells [30,32,33]. Previously, it was determined that *P. gingivalis* loses its ability to surface translocate when the vWF-containing tip protein *mfa5* is deleted [22]. Here, we show that the stress hormone cortisol can stimulate *P. gingivalis* surface translocation and elicit higher expression levels of T9SS-associated genes including *mfa5*.

## 2. Materials and Methods

### 2.1. Bacterial Strains and Culture Conditions

*P. gingivalis* strain W50, strain 381, and derivatives were grown on agar plates containing Todd Hewitt broth (BD Bacto^TM^) supplemented with 5 μg/mL hemin, 1 μg/mL menadione (THBHK) and 5% defibrinated sheep blood (BAPHK) (Northeast Laboratory Services, Winslow, ME, USA), at 37 °C, in an anaerobic chamber (Coy Lab Products, Grass Lake, MI, USA) with an atmosphere containing 5% hydrogen, 10% carbon dioxide, and 85% nitrogen. Broth cultures of *P. gingivalis* were grown anaerobically in THBHK medium. Bacterial growth was then monitored by measuring the optical density at 600 nm and presented as the mean  ±  standard deviations (*n*  =  3).

### 2.2. Stabbing to the Subsurface of Soft Agar Plates (Macroscopic Migration Assay)

To generate plates with surface translocating cells, first, we prepared THBHK soft agar (0.35% agar) plates with and without 2.5% defibrinated sheep blood along with or without cortisol (10 μM) NAD (23 μM), pyruvate (45 mM), or lactate (45 mM). To prepare the inoculum, we grew *P. gingivalis* for 4 days on BAPHK plates in the anaerobic chamber, and then sub-cultured the cells to new BAPHK plates. After overnight incubation, *P. gingivalis* cells were removed from the surface of the agar and suspended in the 500 μL PBS. These high cell density suspensions were then normalized to an OD600 of 50.0 (determined using dilutions). To concentrate the cells further, aliquots (500 μL) of the cell suspensions were centrifuged at 5000× *g* for 15 min. After centrifugation, 400 μL PBS was discarded and the cells were resuspended in the remaining 100 μL of PBS. This concentrated suspension of *P. gingivalis* cells was used for inoculation. The cell resuspension (1 μL) was delivered through the soft agar layer until cells resided at the bottom on the polystyrene plate surface. Plates were observed after 48 of incubation for surface translocation. At least three replicates were used for each set of samples.

### 2.3. Construction of Mutants and in Trans Complementation

*P. gingivalis* strain W50 Δ*mfa5* was generated as previously described [34,35]. Briefly, primers were designed to generate upstream and downstream products of ~1 kb flanking *mfa5*, as well as an erythromycin resistance gene (*ermF*) obtained from plasmid pVA2198. All primers used in this study are listed in Supplementary Table S1. These oligonucleotides were used to prime PCRs using genomic DNA from *P. gingivalis* strain W50 and Phusion high-fidelity PCR master mix with HF buffer according to the manufacturer’s instructions. The products were purified and combined using the NEBuilder HiFi DNA Assembly Master Mix (New England BioLabs, Ipswich, MA, USA) according to the instructions provided by the manufacturer. The final product was mixed with previously frozen cells of *P. gingivalis* and transformed by electroporation. *P. gingivalis* deletion mutants were maintained by supplementing media with 10 µg mL^−1^ erythromycin. Complementation of the *Δmfa5* mutant was performed by inserting mfa5 gene under the control of *groES* promoter region into plasmid pT-COW, generating pT-mfa5. Complemented strain was generated by conjugation as previously described [34,35]. In brief, BAPHK containing tetracycline (1 µg mL^−1^) was used to select for pT-COW containing *P. gingivalis* strains, and gentamicin (200 µg mL^−1^) was used to counterselect the *E. coli* S17-1 donor. Transconjugants were obtained after 7 days of anaerobic incubation. Clones were isolated, verified by PCR, and maintained on BAPHK containing tetracycline (1 µg mL^−1^). Details of bacterial strain and plasmid constructions are provided in Supplementary Table S2.

### 2.4. Microscopy Imaging

Microscopy of translocating cells was performed as previously described with slight modifications [22]. In brief, chamber slides were filled with THBHK soft agar medium (0.35% agar) containing cortisol (10 μM) plus or minus lactate (45 mM). The medium was allowed to solidify for 30 min. To prepare the inoculum, we grew *P. gingivalis* for 4 days on BAPHK plates in the anaerobic chamber, and then sub-cultured the cells to new BAPHK plates. After overnight incubation, *P. gingivalis* cells were removed from the surface of the agar and suspended in the 500 μL PBS. These high cell density suspensions were then normalized to an OD600 of 50.0 (determined using dilutions). To concentrate the cells further, aliquots (500 μL) of the cell suspensions were centrifuged at 5000 × *g* for 15 min. After centrifugation, 400 μL PBS was discarded and the cells were resuspended in the remaining 100 μL of PBS. This concentrated suspension of *P. gingivalis* cells was used for inoculation. Then, a coverslip inoculated with 0.3 μL of *P. gingivalis* cells at the center was inverted and placed onto the chamber filled with medium and mounted with nail polish. Imaging was performed at the interface of the agar medium and coverslip. Phase-contrast images and recorded videos were performed using an inverted Nikon Eclipse Ti microscope system (Nikon, Tokyo, Japan) equipped with a motorized stage (Nikon), an Andor Zyla 5.5 scientific complementary metal oxide semiconductor (sCMOS) camera, a Perfect Focus system, and automated controls (NIS-Elements; Nikon). The microscope was located inside a Coy anaerobic chamber under the conditions described above. Using a Nikon 100 × 1.40-numerical-aperture (NA) lens objective, surface translocation was monitored and recorded for about 20 s for 0 h and 24 h after cell inoculation. Chamber slides were also observed after 48 h of incubation using a Nikon SMZ 745T stereo microscope.

### 2.5. RNA Extraction, and Quantitative PCR (qPCR) Analysis

*P. gingivalis* strain W50 cells were stabbed into pre-reduced BAPHK soft agar with and without 10 μM cortisol in the anaerobic chamber. After 4 h of incubation, cultures were scraped off the plates in the anaerobic chamber, and the RNA extraction was performed using the Direct-zol RNA Miniprep kit (Zymo Research, Irvine, CA, USA), according to the instructions provided by the manufacturer with a slight modification. The qPCR was performed as described previously [36,37]. Briefly, cDNA was generated from 2.5 ng RNA using RNA to cDNA EcoDry premix (Clontech, Mountain View, CA, USA). For relative quantification of desired genes, qPCR was conducted in a total volume of 20 μL containing 1 μL of 1:10 diluted cDNA, a 0.5 μM concentration of each primer (Supplementary Table S1), 6 μL of PCR-grade water, and 10 μL of 2× iQ SYBR green supermix (Bio-Rad, Hercules, CA, USA). Amplification and detection of product were performed using a CFX96 Touch real-time PCR detection system (Bio-Rad), and the cycling conditions were as follows: 95 °C for 3 min and then 39 cycles of 95 °C for 20 s, 55 °C for 20 s, and 72 °C for 20 s. Fluorescence was detected after each cycle. In each experiment, the target and control samples were amplified in the same plate, and the experiments were conducted in triplicate and normalized internally using the average cycle quantification (Cq) value for the reference gene (16S rRNA). To confirm the specificity of the amplified products, automated melting curve analysis was performed.

## 3. Results

### 3.1. Cortisol Promotes Surface Translocation of P. gingivalis in a Concentration-Dependent Manner

**Figure 1:**
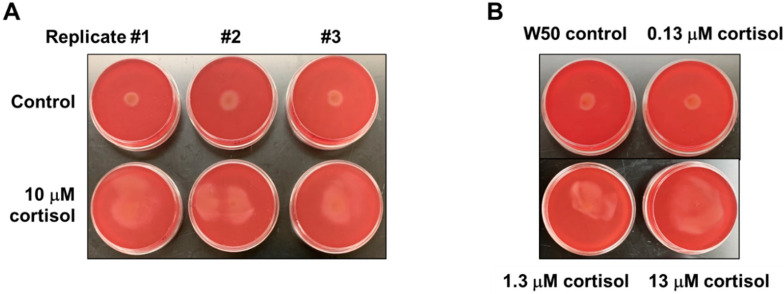
Cortisol promotes surface translocation of *P. gingivalis* in a concentration-dependent manner. Todd Hewitt broth supplemented with 5 μg/mL hemin, 1 μg/mL menadione (THBHK) soft agar (0.35% agar) with 2.5% defibrinated sheep blood was used for the migration assay. Cortisol was added as indicated. (**A**) In the presence of 10 μM cortisol, there is activated movement from the point of inoculation, as opposed to the control. (**B**) *P. gingivalis* cells were stabbed to the subsurface of soft agar plates containing varying cortisol concentrations (0 μM, 0.13 μM, 1.3 μM, and 13 μM), and the plates were observed after 48 h of incubation for surface translocation. Experiments were performed independently at least three times with similar results, and a representative of the results is shown.

Since it has been shown that the stress hormone cortisol can influence the expression level of Type IX Secretion System (T9SS) genes involved in gliding motility in bacteria belonging to the phylum Bacteroidota, we hypothesized that the cortisol could potentially impact the expression level of T9SS-associated genes in *P. gingivalis*. To determine if cortisol alters the migration of *P. gingivalis*, an assay was used that provides an interface between soft agar and a plastic surface. Specifically, *P. gingivalis* was stabbed to the subsurface of soft agar plates until cells resided at the bottom on the polystyrene plate surface, and the plates were observed after 48 h of anaerobic incubation. Interestingly, *P. gingivalis* cells showed activated surface translocation from the point of inoculation in the presence of 10 μM cortisol, as opposed to the control with no cortisol (Figure 1A). To test whether the impact of cortisol on surface translocation of *P. gingivalis* is concentration dependent, cells were stabbed to the subsurface of soft agar plates containing increasing concentrations of cortisol (0 μM, 0.13 μM, 1.3 μM, and 13 μM), and the plates were observed after 48 h of anaerobic incubation for surface translocation. As shown in Figure 1B, higher cortisol concentrations led to more migration of *P. gingivalis* cells. These data support the model that cortisol promotes surface translocation of *P. gingivalis* in a concentration-dependent manner.

### 3.2. Cortisol Promotes P. gingivalis Migration in Rich Medium with the Addition of Lactate or Pyruvate

**Figure 2:**
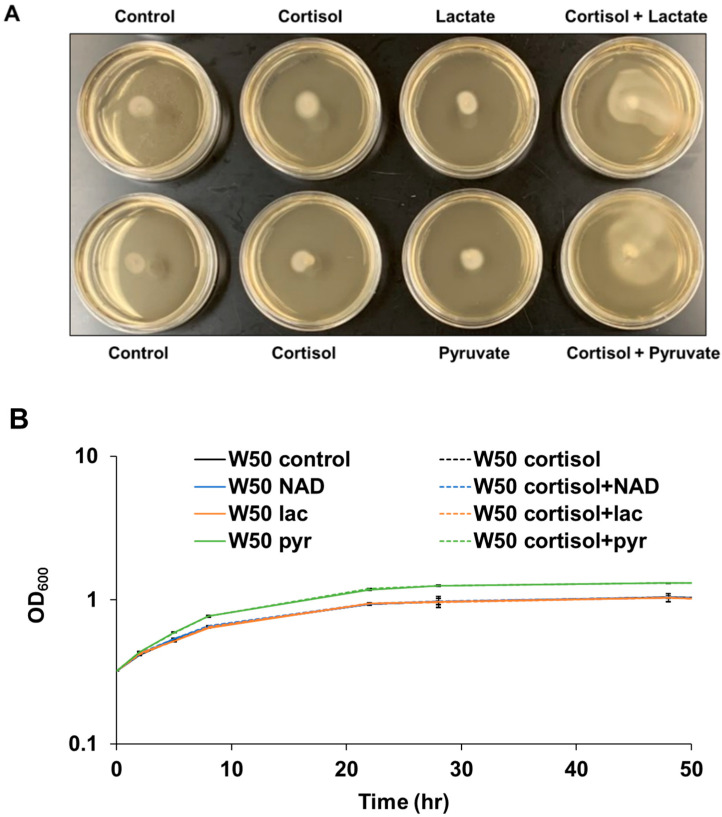
In the absence of blood, cortisol promotes surface translocation of *P. gingivalis* in the rich medium with the addition of lactate or pyruvate. (**A**) THBHK and 0.35% soft agar without sheep blood were used for the surface translocation analysis, and cortisol (10 μM), lactate (45 mM), or pyruvate (45 mM) were added as indicated. Plates were imaged after 48 h of incubation. Experiments were performed independently at least three times with similar results and a representative of the results is shown. (**B**) Growth curve showing that the cortisol has no effect on the growth rate in the THBHK liquid culture. Cortisol (10 μM), NAD (23 μM), pyruvate (45 mM) or lactate (45 mM) added as indicated. Points indicate the mean values, and error bars indicate standard deviations from three technical replicates.

Intriguingly, *P. gingivalis* cells showed little activated surface translocation in THBHK soft agar supplemented with cortisol in the absence of sheep blood as shown in Figure 2A. Previously, it was shown that when combined with protein, exogenous pyruvate and lactate are energy substrates for *P. gingivalis* that affect not only central carbon metabolism, but also the expression of fimbriae, a requirement for surface colonization [27]. Given that red blood cells contain abundant pyruvate and lactate [38,39], and that exogenous pyruvate and lactate influence *P. gingivalis* biofilm development, we hypothesized that cortisol can activate *P. gingivalis* surface translocation in rich medium with the addition of lactate or pyruvate. To determine if these monocarboxylates simply enhance growth, the growth rate of *P. gingivalis* in liquid cultures was tested with the addition of lactate, pyruvate, cortisol, or nicotinamide adenine dinucleotide (NAD) as a control. The data indicate that the cortisol, NAD, and lactate have no effect on the growth rate of *P. gingivalis* in the Todd Hewitt broth supplemented with 5 mg/mL hemin and 1 mg/mL menadione (THBHK) liquid culture, in contrast pyruvate slightly enhanced the growth in the THBHK liquid culture compared to the control (Figure 2B). To observe the surface translocation effect of cortisol, *P. gingivalis* cells were stabbed to the subsurface of soft agar plates containing cortisol, lactate, pyruvate, or NAD. Although cortisol cannot activate surface translocation in the THBHK soft agar supplemented with NAD (Supplementary Figure S1), *P. gingivalis* showed activated surface translocation in THBHK soft agar supplemented with lactate or pyruvate in the presence of cortisol (Figure 2A).

To analyze *P. gingivalis* behavior at the subsurface of soft agar (migration assay) in the presence and absence of cortisol via microscopy, a chamber slide system was used to microscopically observe the cells inside the anaerobic incubator. By placing *P. gingivalis* cells at the interface of a glass coverslip and THBHK soft agar supplemented with lactate in the presence and absence of cortisol, we were able to record short videos of surface translocating cells. Shortly after inoculation, the cells showed little movement regardless of the presence (Supplementary Video S1) or absence (Supplementary Video S2) of cortisol. However, 24 h after inoculation, the cells showed active wriggling motion with cooperative cell-on-cell rolling in the presence of cortisol (Supplementary Video S3) compared to the control (Supplementary Video S4). Overall, the results indicate that cortisol can promote surface translocation of *P. gingivalis* in the rich medium with the addition of sheep blood or exogenous lactate and pyruvate.

### 3.3. Genetic Responses of P. gingivalis Surface Translocation-Associated Genes Are Substantially Altered in the Presence of Cortisol

**Figure 3:**
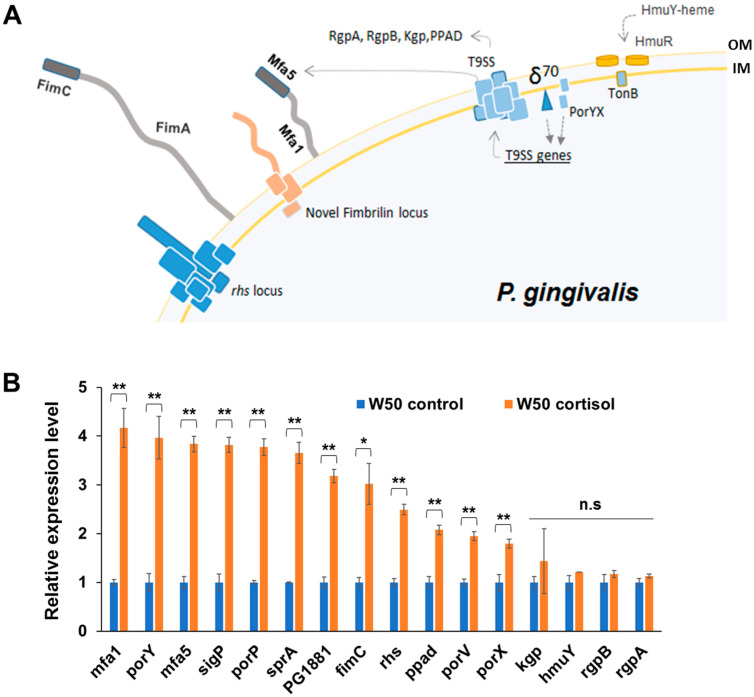
Transcript levels of *P. gingivalis* genes previously shown to be linked to surface translocation are substantially altered in the presence of cortisol. (**A**) Schematic illustration represents surface proteins of *P. gingivalis*. Since T9SS genes have been shown to be up-regulated during surface translocation, we focused on differential expression of the T9SS-associated genes. The number of rectangles reflects the number of proteins involved in a given system. OM; outer membrane, IM; inner membrane. (**B**) Quantitative PCR was used to evaluate differential expression of genes integral to surface translocation when *P. gingivalis* was exposed to cortisol. The results are presented as the relative levels (mean ± S.D. of triplicate determinations) compared with the transcript levels of the strain W50 in the absence of cortisol. The data were analyzed using the Student’s *t*-test. * *p* ≤ 0.05, ** *p* ≤ 0.01, n.s. *p* > 0.05.

To determine if cortisol alters the expression level of genes in *P. gingivalis*, we performed quantitative reverse transcription PCR (qPCR) analysis on RNA samples extracted from subsurface *P. gingivalis* cells in THBHK soft agar supplemented with sheep blood with and without adding cortisol. In particular, we focused on differential expression of the T9SS-associated genes and fimbrial genes since these genes have been shown to be up-regulated during surface translocation of *P. gingivalis* (Figure 3A). Figure 3B represents the relative expression level of target genes of *P. gingivalis* in response to cortisol. The comparison of relative gene expression showed that the *mfa1*, *mfa5* genes encoding the components of minor fimbriae were highly upregulated among various target genes. Further, we determined that cortisol in the medium coincided with the higher transcript levels of *porY*, *sigP*, *porP*, *sprA*, *PG1881*, *fimC*, *rhs*, *ppad*, *porV*, and *porX*, while the expression of gingipains (*kgp*, *rgpA*, *rgpB*) and *hmuY* remained unchanged. Overall, our data show that cortisol can up-regulate the expression level of T9SS-associated and fimbrial genes in *P. gingivalis* cells during migration.

### 3.4. The mfa5 Deletion Mutant Showed Less Surface Translocation Compared with the Parent Strain in the Presence of Cortisol

**Figure 4:**
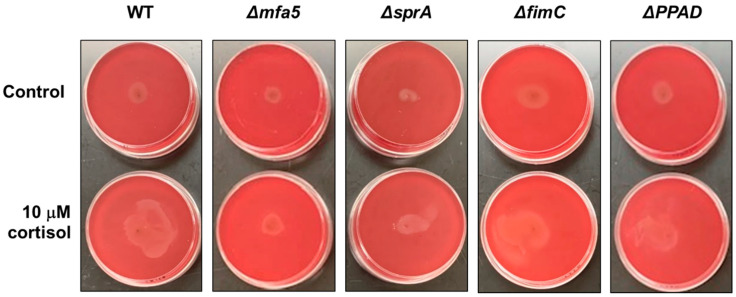
Stabbing of wild-type *P. gingivalis* strain 381 and its derivatives into blood agar plate in the absence and presence of cortisol. THBHK soft agar (0.35% agar) with 2.5% defibrinated sheep blood were used for the surface translocation analysis, cortisol added as indicated. Plates were imaged after 48 h of incubation for migration from the point of inoculation. Experiments were performed independently at least three times with similar results and a representative of the results is shown.

**Figure 5:**
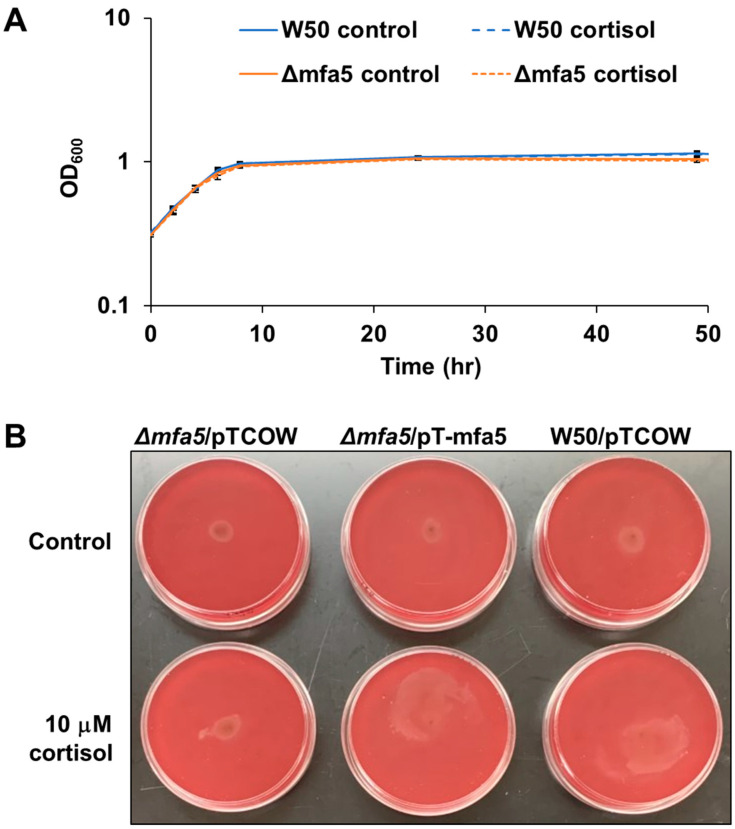
The surface translocation defects of the *mfa5* deletion mutant were restored by complementation. (**A**) Wild-type W50 and the *Δmfa5* mutant were grown in THBHK liquid media in the presence and absence of cortisol. Points indicate the mean values, and error bars indicate standard deviations from three replications. (**B**) When the *mfa5* deletion mutant was complemented by a plasmid (pT-mfa5), surface translocation was restored. THBHK soft agar (0.35% agar) with 2.5% defibrinated sheep blood was used for the migration assay, and cortisol was added as indicated. Plates were imaged after 48 h of incubation for surface translocation. Experiments were performed independently at least three times with similar results, and a representative of the results is shown.

Previously, it was shown that the *P. gingivalis* strain 381 can surface translocate when sandwiched between two surfaces [22]. To identify the underlying mechanism controlling surface translocation in the presence of cortisol, we used four mutant strains in *P. gingivalis* strain 381, specifically, strains with deletions in *mfa5*, *sprA* (or *sov*), *fimC*, or *ppad*, since the qPCR data showed upregulated expression level of those genes in the presence of cortisol. The *sprA* gene encodes a major component of the envelope spanning T9SS multiprotein complex [40]. SprA is necessary for secretion of various factors including a peptidylarginine deiminase (PPAD) which converts charged arginine residues within peptides to citrulline. FimC is a tip protein of the major fimbriae. Studies have shown that this protein promotes surface attachment and biofilm formation. Lastly, Mfa5 is incorporated into the polymerization process of minor fimbriae affecting the incorporation of other accessory subunits [29]. As shown in Figure 4, the *mfa5* deletion mutant showed less surface translocation compared with the wild-type 381 strain and other mutant strains in the presence of cortisol. To evaluate the effect of the loss of *mfa5* in the *P. gingivalis* strain W50, we generated a W50 *mfa5* deletion mutant and compared the mutant with the parent strain. Addition of cortisol (10 μM) showed no effect on the growth rate of *P. gingivalis* strain wild-type W50 and *mfa5* deletion mutant in the THBHK liquid culture (Figure 5A), and the surface translocation defects of the *mfa5* deletion mutant were restored by complementation (Figure 5B). Overall, our data support the model that cortisol can upregulate the expression levels of T9SS-associated genes including *mfa5* and activate the surface translocation of *P. gingivalis*.

## 4. Discussion

Studies have shown that both bacterial and human hormones are important mechanisms for host–microbial interaction and the interplay is highly complex [5,41]. For instance, host hormones can affect bacterial gene expression, which in turn can impact the host’s innate immune response. A few recent studies have shown that stress hormones, including cortisol, significantly affect endogenous periodontal pathogens [3,4,5]. Cortisol levels have been shown to be higher in saliva and serum in subjects with periodontal disease [6,7]. While salivary cortisol levels have been reported to range between 0.02 ± 0.008 μM for healthy adults and the levels can increase to greater than 0.15 μM in periodontitis patients [6,42,43]; normal cortisol levels in human serum have been reported to have a broad range between 0.14 to 0.55 μM and can increase to greater than 1.24 μM in times of stress [44]. In this study, we found that *P. gingivalis* does not migrate far from the point of inoculation when exposed to relatively low levels of cortisol (0.13 μM), yet higher stress-level concentrations of cortisol (1.3 μM) promoted migration, and the response can be elicited in a dose dependent manner. Importantly, the impact of cortisol on surface translocation was only observed under certain growth conditions. As noted above, *P. gingivalis* is highly proteolytic, obtaining its nutrients from protein. To generate peptides for uptake, proteolytic enzymes are released from the cells into the environment either on outer membrane vesicles or extended out into the surrounding from the cell surface via nanotubule protrusions [45,46]. The need for *P. gingivalis* cells to spread proteases seems especially true in our sandwich model/migration assay where *P. gingivalis* is growing on a substratum and is limited for substrate. Additionally, of significance to this study, many of these secreted enzymes lyse erythrocytes. Since the primary function of erythrocytes is to bind and transport oxygen, we typically think of *P. gingivalis* lysing erythrocytes within bleeding pockets to obtain heme/iron. However, because erythrocytes lack mitochondria and rely completely on the glycolytic pathway to generate ATP, it follows that lysis of erythrocytes would not only releases hemoglobin (iron source), but also glycolytic metabolites, such as lactate and pyruvate into the immediate surroundings. Recently, a metabolic coupling system in *P. gingivalis* was identified that enables the utilization of protein coupled with exogenous pyruvate and lactate [27]. This information, combined with the fact that oral streptococci are known to be able to produce high levels of lactate, we hypothesized that select metabolites, in particular, those that are contained within erythrocytes (or produced by other bacteria), were potentially the underlying reason why the impact of cortisol on surface migration was only detected when sheep blood was provided. Indeed, our study discovered that under our assay conditions, sheep blood could be replaced with lactate or pyruvate, yet not NAD (Figure 2A and Figure S1). It is likely that other metabolites within erythrocytes or produced by other oral microbiota also impact *P. gingivalis* surface translocation, yet these findings provide a foundation for further studies.

In regard to the fimbrial tip protein Mfa5, in this study and in a previous study, it was confirmed that *P. gingivalis* loses its ability to surface translocate when *mfa5* is deleted [22]. We have also shown that exposure to cortisol in the presence of sheep blood can result in higher *mfa5* transcript levels, suggesting that cortisol impacts *mfa5* expression, but the effect is context dependent. Importantly, disruption of the T9SS in *P. gingivalis* inhibits export of Mfa5 to the outer membrane, and Mfa5 is unique among the fimbrial proteins in that it is transported via the T9SS and it is a large multi-domain protein, one of which is a von Willebrand factor (vWF) domain. Since Mfa5 has a vWF domain and shows weak homology (21% identity) with RemA, a mobile cell surface adhesin [47], there is a high possibility that Mfa5 may have a similar function in *P. gingivalis* as a surface translocation adhesin. Additionally, of interest was the discovery that the lipoprotein encoded by PG1881, which is described as a structural homolog to FimA and Mfa1 in silico [48,49], was also upregulated in the presence of cortisol. This pilin-forming lipoprotein was found to be expressed at high levels during migration [22]. Experiments to identify the role of PG1881 in adherence and surface translocation are on-going.

Importantly, since SprA is a central component of the T9SS and essential for type IX secretion, it was expected that the *sprA* deletion mutant would not migrate, yet the mutant showed a low level of migration compared to the parent strain in the presence of cortisol. The simplest explanation for these results is that some T9SS cargo proteins can be secreted at a certain level via another secretion system, yet further studies are needed to clarify these findings. Lastly, one of the most remarkable discoveries of this study was that genes that encode T9SS structural proteins, including PorP, PorV, and SprA along with the PorXY two component system (TCS) and the extracytoplasmic function (ECF) sigma factor SigP that regulates the transcriptional level of T9SS genes were upregulated in the presence of cortisol. Although it is intriguing to think that the PorXY TCS may be involved in sensing cortisol and directly regulating gene expression, the *mfa5* deletion mutant could be complemented in *trans* with a non-native promoter, suggesting that impact of cortisol is an indirect effect on *mfa5* transcription. Further studies are required to determine the cortisol sensing and signal transduction mechanisms.

## 5. Conclusions

Our working model is that the increased level of cortisol under conditions of stress can elicit changes in *P. gingivalis* gene expression and activate surface translocation; in particular, exposure to cortisol in the presence of pyruvate or lactate elicits higher expression levels of T9SS-associated genes. Since the von Willebrand factor type A domain-containing proteins, such as Mfa5 at the tips of fimbrial fibers, play important roles in adhesion and surface migration, there is a high possibility that Mfa5 plays a central role in *P. gingivalis* surface translocation. Although the underlying mechanisms for translocation in the presence of cortisol remains to be determined, we propose that our findings open a new avenue for studying how *P. gingivalis* and other subgingival members of the oral Bacteroidota adjust their lifestyle in response to host hormones.

## Data Availability

Not applicable.

## Supplementary Materials

The following supporting information can be downloaded at: https://www.mdpi.com/article/10.3390/pathogens11090982/s1, Figure S1: In the absence of blood, cortisol plates showed the same surface translocation of *P. gingivalis* compared to the control regardless of adding NAD; Table S1: Primers used in this study; Table S2: Strains and plasmids used in this study; Video S1: Shortly after inoculation, the cells showed little movement in the presence of cortisol; Video S2: Shortly after inoculation, the cells showed little movement in the absence of cortisol; Video S3: 24 h after inoculation, the cells showed active wriggling motion with cooperative cell-on-cell rolling in the presence of cortisol; Video S4: 24 h after inoculation, the cells showed little movement in the absence of cortisol. References [22,50,51] are cited in the supplementary materials.
