# Supplementary material for: Cortisol Promotes Surface Translocation of Porphyromonas gingivalis
Source: Pathogens. 2022 Aug 27;11(9):982. doi: 10.3390/pathogens11090982 (PMC9505793; doi:10.3390/pathogens11090982)
Supplement: Supplementary file 1 [file pathogens-11-00982-s001.zip › Figure S1, Tables S1 and S2.pdf]

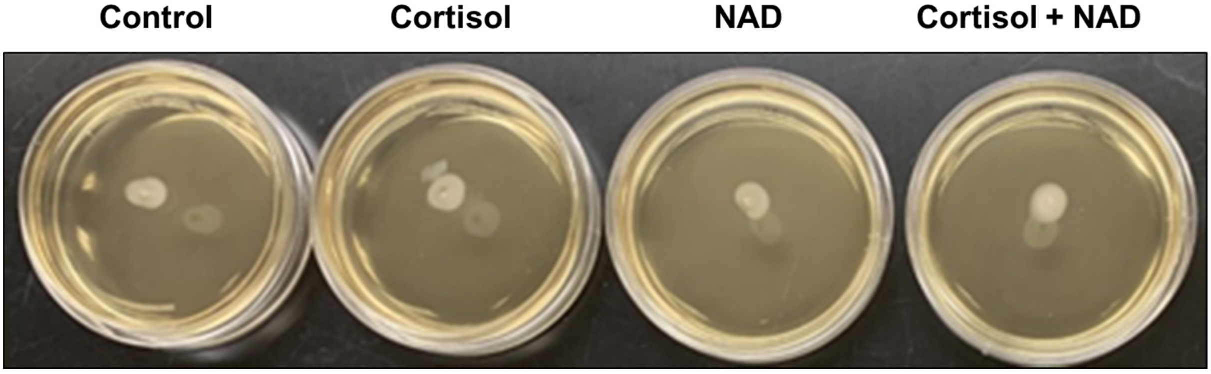

Supplementary Figure S1. In the absence of blood, cortisol plates showed the same surface translocation of *P. gingivalis* compared to the control regardless of adding NAD. THBHK and 0.35% agar was used for the surface translocation analysis, and 10 µM cortisol or 23 µM NAD was added as indicated. Plates were observed after 48 hours of incubation for surface translocation.

Supplementary Table S1. Primers used in this study

Name	Sequence (5'-3')	Purpose
pUC19_FW	GCGGGCCTCTTCGCTATTAC	Sequencing of *Δmfa5*::Erm on plasmid
pUC19_RV	GATGCGGTATTTTCTCCTTAC	Sequencing of *Δ mfa5*::Erm on plasmid
mfa5_delF1_fwd	TTGTAAAACGACGGCCAGTGAATTC GATGATTTCACCAAAACG	Deletion of *Δ mfa5*::Erm
mfa5_delF1_rev	TCTTTTTTGTCATTATTATTACCTCGT TAGTTACTAC	Deletion of *Δ mfa5*::Erm
mfa5_delF2_fwd	GAGGTAATAATAATGACAAAAAAG AAATTGCC	Deletion of *Δ mfa5*::Erm
mfa5_delF2_rev	CATTATATCAACCTACGAAGGATGA AATTTTTC	Deletion of *Δ mfa5*::Erm
mfa5_delF3_fwd	TCATCCTTCGTAGGTTGATATAATGG TACCCC	Deletion of *Δ mfa5*::Erm
mfa5_delF3_rev	CTATGACCATGATTACGCCAAGCTT GATAGATGATCTCGCCGTTG	Deletion of *Δ mfa5*::Erm
W50_mfa5_seq_FW	TTGTTATATGCCTCGTTGCTAAC	Sequencing of *Δmfa5*::Erm on gDNA
W50_mfa5_seq_RV	AGCAGCGGTAGTTTTTATCTC	Sequencing of *Δmfa5*::Erm on gDNA
16s rRNA_FW	TGTTACAATGGGAGGGACAAAGGG	qPCR
16s rRNA_RV	TTACTAGCGAATCCAGCTTCACGG	qPCR
mfa1_FW	GGAGAACGTCGCCAATACCT	qPCR
mfa1_RV	AATCAGCCAAAGTCGGCACT	qPCR
porY_FW	ACCGACTGCGTATGATAGCC	qPCR
porY _RV	AAAGTGCTATGCCGATGACC	qPCR
mfa5_FW	TTGTGCCGATTGTGGTATGT	qPCR
mfa5_RV	TAAACTCCCGTAGGCTGTGC	qPCR
sigP_FW	TGGCGGAGGATATTTTTCAG	qPCR
sigP _RV	GACTGCAATCTGGCTCCTTC	qPCR

porP_FW	TACCAAAGTGAGCGGAAAGG	qPCR
porP_RV	AACGAATAAATGGAGCGGATA	qPCR
sprA_FW	GCAAAAGGAGGGCAAAAGA	qPCR
sprA_RV	TGACAAGCGGATGGTTCATA	qPCR
PG1881_FW	ACAGCATCAAGAAGGCGAAA	qPCR
PG1881_RV	GCGGAACAATCGGTGAAC	qPCR
fimC_FW	CGCCAAGATCACACTGGAC	qPCR
fimC_RV	AAGATCGCAGGCATTCTCC	qPCR
ppad_FW	TAGGTGTCAAAGGGGCTTCA	qPCR
ppad_RV	CGGCGAGATAGTAGCATTGG	qPCR
porV_FW	GGGAACAGACGAGAATCAGG	qPCR
porV_RV	TGAGCGGATGTAACGCAGT	qPCR
porX_FW	AGAAGCCGTGCAAAATAACG	qPCR
porX_RV	ACCGGTTTGATGAGGTAGTCC	qPCR
kgp_FW	CGCAGGGTCAGAAAGTAACG	qPCR
kgp_RV	ATCGGCATCCAACAAGAACT	qPCR
hmuY_FW	CCGAAGCAGTAACCAAAACC	qPCR
hmuY_RV	GCACCACCTTTTCCCTTACC	qPCR
rgpB_FW	CGAGGTGAAAGTGGCAGAAG	qPCR
rgpB_RV	TGAGAAACGACCGATGAAGA	qPCR
rgpA_FW	TCCTTCTTGTTCCCCTACCA	qPCR
rgpA_RV	CACCGCTACCCATCTTCTTC	qPCR
groES_fwd	GGATCCGCATGCCCCATTGGATAGATGCCCTGC	Cloning of mfa5 with groES promoter on pT-COW
groES_rev	GTTTCATCATTGTTGCTTGGTTTGTTATTG	Cloning of mfa5 with groES promoter on pT-COW

mfa5_fwd	CCAAGCAACAATGATGAAACGATAT ACAATAATTC	Cloning of mfa5 with groES promoter on pT-COW
mfa5_rev	CGGCCGGTCGACCCCTTAATACTCA ACAAGCACC	Cloning of mfa5 with groES promoter on pT-COW
W50_mfa5_seq1	TTCAAGCTGAAATTGTATACC	Sequencing of groES-*mfa5* on pT-COW
W50_mfa5_seq2	CAATCCATCTACACCTCTTAC	Sequencing of groES-*mfa5* on pT-COW
W50_mfa5_seq3	TTCCCACGTCCGACGGTTAAG	Sequencing of groES-*mfa5* on pT-COW
W50_mfa5_seq4	CGGGCTATAATCAAAATGTAGGG	Sequencing of groES-*mfa5* on pT-COW
pTCOW_seq_FW	CAGTGAGGATATTGACGCTTATTTC G	Sequencing of groES-*mfa5* on pT-COW
pTCOW_seq_RV	CGCATTCACAGTTCTCCGCAAG	Sequencing of groES-*mfa5* on pT-COW

Supplementary Table S2. Strains and plasmids used in this study

**Strain (relevant genotype)**	**Source or reference**
***P. gingivalis* strains**	
W50	Christian Mouton, Laval University, Quebec City, Canada
381	H. Kuramitsu, State University of Buffalo, Buffalo, NY
*Δ*mfa5::Erm (Em^r) in strain 381	Moradali *et al.*[1]
*Δ*sprA::Erm (Em^r) in strain 381	Moradali *et al.*[1]
*Δ*fimC::Erm (Em^r) in strain 381	Moradali *et al.*[1]
*Δ*ppad::Erm (Em^r) in strain 381	Moradali *et al.*[1]
*Δ*mfa5::Erm (Em^r) in strain W50	This study
***E. coli* strain**	
NEB 5α	NEB
S17-1	Matsumoto-Mashimo *et al.*[2]
**Plasmids**	
pUC19	NEB
pT-COW (Amp^R and Tc^R in *E. coli*; Tc^R in *P. gingivalis*; Mob^+ Rep^+)	Gardner *et al.*[3]
pT-mfa5 (Amp^R and Tc^R in *E. coli*; Tc^R in *P. gingivalis*; Mob^+ Rep^+)	This study

References

1. Moradali, M.F.; Ghods, S.; Angelini, T.E.; Davey, M.E. Amino acids as wetting agents: surface translocation by *Porphyromonas gingivalis*. *ISME J* **2019**, *13*, 1560-1574, doi:10.1038/s41396-019-0360-9.
2. Matsumoto-Mashimo, C.; Guerout, A.M.; Mazel, D. A new family of conditional replicating plasmids and their cognate *Escherichia coli* host strains. *Res Microbiol* **2004**, *155*, 455-461, doi:10.1016/j.resmic.2004.03.001.
3. Gardner, R.G.; Russell, J.B.; Wilson, D.B.; Wang, G.R.; Shoemaker, N.B. Use of a modified Bacteroides-Prevotella shuttle vector to transfer a reconstructed beta-1,4-D-endoglucanase gene into Bacteroides uniformis and Prevotella ruminicola B(1)4. *Appl Environ Microbiol* **1996**, *62*, 196-202, doi:10.1128/AEM.62.1.196-202.1996.
